# Understanding the link between mental health disorders and professional quality of life among humanitarian workers: A cross-sectional study from the Thai-Myanmar border

**DOI:** 10.1016/j.puhip.2025.100694

**Published:** 2025-12-11

**Authors:** Roshan Kumar Mahato, Naw Lar Paw, Kyaw Min Htike, Rajitra Nawawonganun

**Affiliations:** Department of Health Management Innovative Technology, Faculty of Public Health, Khon Kaen University, Khon Kaen, Thailand

**Keywords:** ProQOL, Humanitarian aid workers

## Abstract

**Objectives:**

The study aimed to explore the relationship between mental health disorders and professional quality of life (ProQOL) among humanitarian aid workers (HAWs) along the Thai-Myanmar border focusing on compassion satisfaction, burnout and secondary traumatic stress (STS).

**Study design:**

Cross-sectional study.

**Methods:**

Data was collected through surveys using validated tools to measure compassion satisfaction, burnout, and secondary traumatic stress. An independent *t*-test and ANOVA were used to compare groups. Linear regression models were applied to assess the relationships between social support, stress, and job outcomes. Pearson correlation was used to explore the associations between key variables, with significance set at P < 0.05.

**Results:**

The mean scores were 34.87 ± 6.55 for compassion satisfaction, 24.68 ± 5.31 for burnout and 25.16 ± 6.19 for STS. Higher stress, depression and PTSD significantly increased burnout and STS. Quality of life (QOL) was positively linked with compassion satisfaction and negatively associated with burnout and STS. Multiple linear regression showed that family support (AMD: 1.524, 95 % CI: 1.14–1.91) and QOL (AMD: 0.088, 95 % CI: 0.04–0.14) predicted higher compassion satisfaction. Burnout was negatively associated with family support (AMD: 0.951, 95 % CI: 1.25 to −0.65) and QOL (AMD: 0.054, 95 % CI: 0.09 to −0.01) however positively linked to stress and PTSD.

**Conclusion:**

This study provided the mental health challenges of HAWs, emphasizing protective and risk factors that can inform targeted interventions to enhance their well-being in high-risk settings.

## 1 Introduction

Humanitarian aid workers, including professionals and volunteers with NGOs and UN agencies, support crisis victims through healthcare, protection, and education services [1]. Since the 2021 Myanmar coup, humanitarian aid efforts along the Thai-Myanmar border have intensified, with NGOs, CSOs, and CBOs working to meet the needs of forcibly displaced people (FDPs) and migrants in health, education, and emergency response [2]. As many activists and families fleeing Myanmar's instability have sought refuge in Thailand, the Royal Thai Government, UNHCR, and NGOs have expanded their assistance efforts [3]. This heightened demand for aid services has increased stress on workers, underscoring the need to understand their professional quality of life.

The Thai-Myanmar border is a challenging humanitarian setting, where aid workers face limited resources, political instability, and safety risks as they respond to vulnerable populations’ needs [4]. These conditions placed significant emotional and physical demands on aid workers affecting their well-being and job satisfaction. The ProQOL Scale assesses both positive (Compassion Satisfaction) and negative (Compassion Fatigue) impacts of working with trauma survivors. Compassion satisfaction reflects the joy of helping others, while compassion fatigue includes burnout symptoms, such as exhaustion and frustration, as well as secondary traumatic stress from work-related trauma [5]. Studies have shown that anxiety and depression can negatively impact ProQOL, increasing compassion fatigue and burnout [6] while effective coping can enhance well-being positively influencing ProQOL [7]. This study aimed to explore the link between mental health disorders and professional quality of life among humanitarian aids workers along the Thai-Myanmar border.

## 2 Methods

### 2.1 Study area

The study was conducted in Thai-Myanmar border specifically within the provinces of Tak, Mae Hong Son, Ratchaburi and Kanchanaburi from October 2023 to September 2024. These areas were chosen due to their role as central hubs for humanitarian assistance provided to refugees and migrants with numerous NGOs, CSOs and UN agencies active in these regions responding to recent emergencies and supporting vulnerable populations.

### 2.2 Study Design

This research adopted an analytical cross-sectional design to assess the job-related professional quality of life of humanitarian aid workers stationed along the Thai-Myanmar border. It also examined the effects of various social determinants on their overall well-being.

### 2.3 Sample size and sampling

The sample size was calculated using the multiple logistic regression formula by Hsieh, Bloch, & Larsen, 1998 [8]. The initial estimate was approximately 212 participants. To prevent overfitting, adjustments were applied, including a Rho Square value of 0.70 and a variance inflation factor (VIF) of 3.33, resulting in a final sample size of approximately 424 participants. Sampling was conducted using a probability proportional to size (PPS) method across the four selected provinces with districts as the sampling units. Within each district, participants were randomly selected based on the size of the target population in each group.

### 2.4 Data collection

Data were collected using a structured, multilingual questionnaire translated into Thai, Myanmar, and Karen through forward-backward translation. The questionnaire included multiple validated tools: demographic and health information; family functioning (Family APGAR) [9]; perceived social support (MPSS) [10]; depression (Major Depression Inventory) [11]; stress (Perceived Stress Scale) [12]; PTSD symptoms (SPRINT) [13]; self-esteem (Rosenberg Self-Esteem Scale) [14]; and quality of life (WHOQOL-BREF) [15]. Each tool included standardized scoring systems to assess the respective domains, ensuring comprehensive coverage of psychosocial, emotional, and quality of life factors relevant to humanitarian aid workers.

### 2.5 Outcome variable measurement

The Professional Quality of Life Scale was used to assess compassion satisfaction, burnout, and secondary traumatic stress with scores ranging from 10 to 50 for each subscale [5]. A pre-test involving 20 % of participants demonstrated strong reliability across all scales, with Cronbach's alpha values ranging from 0.79 to 0.94. Content validity was ensured through expert review and data were gathered via face-to-face interviews.

### 2.6 Data analysis

Data were entered into Microsoft Excel 365 and analyzed using STATA version 18.0 (College Station, Texas 77845 USA). Categorical variables were summarized as frequencies and percentages, while continuous variables were reported as means, standard deviations, medians and ranges. Inferential statistics included independent t-tests for comparing two groups, and ANOVA for comparing multiple groups were performed. Linear regression was used to assess the relationships between social support, stress and job outcomes. Pearson correlation coefficients measured the strength and direction of associations between key variables. A significance level of p < 0.05 was used for all tests. Missing data were addressed through complete case analysis.

## 3 Results

### 3.1 Descriptive statistics (mean ± SD) for compassion satisfaction, burnout and secondary traumatic stress

The mean Compassion Satisfaction score among humanitarian aid workers was 34.87 ± 6.55 indicating a moderate level of satisfaction derived from helping others. The Burnout score had a mean of 24.68 ± 5.31 suggesting a moderate risk of emotional exhaustion and work-related stress. Meanwhile, the Secondary Traumatic Stress score averaged 25.16 ± 6.19 reflecting the potential emotional impact of indirect exposure to trauma through their work (Table 1).

**Table 1:** Descriptive statistics (mean ± SD) for compassion satisfaction, burnout and secondary traumatic stress (n = 424).

Domain of ProQoL	Mean	SD
**Compassion satisfaction**	34.87	±6.55
**Burnout**	24.68	±5.31
**Secondary Traumatic Stress**	25.16	±6.19

### 3.2 Factors influencing job-related outcomes for humanitarian aid workers

The factors influencing job-related outcomes for humanitarian aid workers specifically focusing on compassion satisfaction, burnout and secondary traumatic stress. Support from family, stress and quality of life were significant predictors of compassion satisfaction. High family support was strongly associated with increased compassion satisfaction with an AMD of 1.524 (95 % CI: 1.14 to 1.91). Conversely, higher stress levels were negatively associated with compassion satisfaction (AMD: 0.160, 95 % CI: 0.29 to −0.03) suggesting that increased stress reduced the satisfaction derived from their work. Quality of life also positively correlated with compassion satisfaction with an AMD of 0.088 (95 % CI: 0.04 to 0.14) indicating that a higher quality of life enhanced satisfaction levels (Table 2).

**Table 2:** Bivariate and multivariable analysis of factors associated with Compassion Satisfaction among Humanitarian Aid Workers (n = 424).

Factors	Mean (±SD)	Compassion satisfaction					
		Crude Mean Diff.	95 % CI	P-value	AMD	95 % CI	P-value
**Age (Year)**	33.76 (±8.78)	0.051	−0.02 to 0.122	0.160			
**Family Relationship score**	6.49 (±2.65)	0.418	0.18 to 0.65	<0.001			
**Support received from family Score**	4.91 (±1.49)	1.807	1.42 to 2.19	<0.001			
**Support received from friends score**	4.55 (±1.18)	1.524	1.01 to 2.04	<0.001	1.524	1.14 to 1.91	<0.001
**Support received from significant other Score**	4.05 (±1.17)	1.427	0.91 to 1.94	<0.001			
**Depression Score**	12.59 (±9.65)	−0.142	−0.21 to −0.08	<0.001			
**Stress Score**	17.55 (±4.80)	−0.343	−0.47 to −0.22		−0.160	−0.29 to −0.03	0.017
**PTSD Score**	9.40 (±6.64)	0.002	−0.09 to 0.10	0.970			
**Self-esteem score**	15.38 (±1.81)	0.392	0.05 to 0.74	0.026			
**Quality of life Score**	91.58 (±12.34)	0.167	0.12 to 0.22	<0.001	0.088	0.04 to 0.14	<0.001

Similarly, burnout was significantly influenced by family support, stress, PTSD and quality of life. Family support was inversely related to burnout with an AMD of −0.951 (95 % CI: 1.25 to −0.65) meaning that greater family support reduced burnout levels. Stress was positively associated with burnout (AMD: 0.310, 95 % CI: 0.20 to 0.42) indicating that high-stress levels lead to increased burnout. PTSD also showed a positive association with burnout with an AMD of 0.090 (95 % CI: 0.14 to 0.17) suggesting that PTSD symptoms contributed to burnout in this workforce. Lastly, quality of life had a protective effect against burnout with a negative AMD of −0.054 (95 % CI: 0.09 to −0.01) (Table 3).

**Table 3:** Bivariate and multivariable analysis of factors associated with Burnout among Humanitarian Aid Workers (n = 424).

Characteristics	Mean (±SD)	Burnout					
		Crude Mean Diff.	95 % CI	P-value	AMD	95 % CI	P-value
**Age**	33.76 (±8.78)	0.021	−0.04 to 0.08	0.474			
**Family Relationship**	6.49 (±2.65)	−0.385	−0.57 to −0.20	<0.001			
**Support received from family**	4.91 (±1.49)	−1.285	−1.60 to −0.97	<0.001			
**Support received from friends**	4.55 (±1.18)	−0.922	−1.35 to −0.50	<0.001	−0.951	−1.25 to −0.65	<0.001
**Support received from significant other**	4.05 (±1.17)	−1.10	−1.52 to −0.67	<0.001			
**Depression**	12.59 (±9.65)	0.186	0.14 to 0.24	<0.001			
**Stress**	17.55 (±4.80)	0.486	0.39 to 0.58	<0.001	0.310	0.20 to 0.42	<0.001
**PTSD**	9.40 (±6.64)	0.269	0.20 to 0.34	<0.001	0.090	0.14 to 0.17	0.021
**Self-esteem**	15.38 (±1.81)	−0.272	−0.55 to 0.01	0.056			
**Quality of life**	91.58 (±12.34)	−0.160	−0.20 to −0.12	<0.001	−0.054	−0.09 to −0.01	0.009

Furthermore, Secondary traumatic stress (STS) from work was significantly associated with both stress and PTSD. Higher stress was associated with an increase in STS, with an AMD of 0.215 (95 % CI: 0.09 to 0.34) highlighting that more stressful conditions elevated STS levels. PTSD showed an even stronger positive association with STS with an AMD of 0.357 (95 % CI: 0.27 to 0.45) indicating that PTSD symptoms significantly contributed to STS among these workers (Table 4).

**Table 4:** Bivariate and multivariable analysis of factors associated with Secondary Traumatic Stress among Humanitarian Aid Workers (n = 424).

Characteristics	Mean (±SD)	Secondary Traumatic Stress					
		Crude Mean Difference	95 % CI	P-value	AMD	95 % CI	P-value
**Age**	33.76 (±8.78)	−0.019	−0.09 to 0.05	0.586			
**Family Relationship**	6.49 (±2.65)	−0.156	−0.38 to 0.07	0.169			
**Support received from family**	4.91 (±1.49)	−0.092	−0.49 to 0.31	0.649			
**Support received from friends**	4.55 (±1.18)	0.0001	−0.50 to 0.50	0.999			
**Support received from significant other**	4.05 (±1.17)	−0.236	−0.74 to 0.27	0.359			
**Depression**	12.59 (±9.65)	0.194	0.14 to −0.25	<0.001			
**Stress**	17.55 (±4.80)	0.467	−0.35 to −0.58	<0.001	0.215	0.09 to 0.34	<0.001
**PTSD**	9.40 (±6.64)	0.437	0.36 to 0.52	<0.001	0.357	0.27 to 0.45	<0.001
**Self-esteem**	15.38 (±1.81)	−0.019	−0.35 to 0.31	0.909			
**Quality of life**	91.58 (±12.34)	−0.117	−0.16 to −0.07	<0.001			

### 3.3 Correlation of various factors with the subscale of ProQoL

The correlation analysis revealed several important relationships between various factors and the professional quality of life among humanitarian aid workers. Compassion satisfaction was positively correlated with family support (r = 0.411), support from friends (r = 0.274) and significant others (r = 0.255) suggesting that greater social support enhances job satisfaction. Additionally, quality of life was positively correlated with compassion satisfaction (r = 0.315) indicating that a higher quality of life contributes to greater job satisfaction. In contrast, depression (r = −0.210) and stress (r = −0.251) were weakly negatively correlated with compassion satisfaction, meaning that higher levels of these factors reduce job satisfaction. For burnout, a moderate positive correlation was found with stress (r = 0.440) and weak positive correlations were observed with depression (r = 0.339) and PTSD (r = 0.336) suggesting that higher stress, depression and PTSD levels contribute to burnout. Conversely, family support (r = −0.360), friend support (r = −0.204) and support from significant others (r = −0.241) were weakly negatively correlated with burnout, indicating that social support helps alleviate burnout. Quality of life (r = −0.372) was also negatively correlated with burnout suggesting that higher quality of life reduces burnout. Regarding STS, weak positive correlations were found with stress (r = 0.363), PTSD (r = 0.469) and depression (r = 0.303) highlighting that higher levels of stress, PTSD and depression contribute to STS. Finally, quality of life was weakly negatively correlated with STS (r = −0.233) suggesting that a better quality of life may help reduce secondary traumatic stress (Table 5).

**Table 5:** Correlation analysis of various factors with compassion satisfaction, burnout and secondary traumatic stress among humanitarian aid workers.

Factors	Compassion satisfaction		Burnout		Secondary Traumatic Stress	
	Correlation Coefficient	P-value	Correlation Coefficient	P-value	Correlation Coefficient	P-value
**Age**	0.068	0.160	0.035	0.474	−0.027	0.586
**Family Relationship**	0.169	<0.001	−0.192	<0.001	−0.067	0.169
**Support received from family**	0.411	<0.001	−0.360	<0.001	−0.022	0.649
**Support received from friends**	0.274	<0.001	−0.204	<0.001	N/A	N/A
**Support received from significant other**	0.255	<0.001	−0.241	<0.001	−0.045	0.359
**Depression**	−0.210	<0.001	0.339	<0.001	0.303	<0.001
**Stress**	−0.251	<0.001	0.440	<0.001	0.363	<0.001
**PTSD**	0.002	0.970	0.336	<0.001	0.469	<0.001
**Self-esteem**	0.108	0.026	−0.093	0.056	−0.006	0.909
**Quality of life**	0.315	<0.001	−0.372	<0.001	−0.233	<0.001

## 4 Discussion

The results showed that family support and strong family relationships played a positive role in enhancing compassion and reducing burnout among humanitarian aid workers. This suggests that family-based or community-oriented interventions could be crucial in supporting aid workers’ mental health and job satisfaction. Additionally, while support from friends and significant others had a somewhat weaker effect than family support, it still underscored the importance of broader social support networks for promoting the well-being of aid workers. Similarly, a study from Spain found that perceived social support significantly affected all three dimensions of professional quality of life, with the greatest impact observed on burnout. This highlights the critical role of social support in mitigating burnout and promoting overall well-being in high-stress professions [16]. A study from China found that both informal and formal support was positively linked to compassion satisfaction and negatively correlated with job burnout. Informal support, such as personal connections, was particularly effective in enhancing job satisfaction and reducing burnout. At the same time, formal support, like organizational resources, also helped improve well-being and lower burnout levels [17].

Mental health emerged as a significant predictor of job outcomes with depression, stress and PTSD symptoms directly impacting compassion satisfaction, burnout and secondary traumatic stress (STS). The findings showed that untreated mental health issues posed a substantial risk for burnout and STS, especially in cases of severe symptoms. This underscored the need for mental health screening and interventions within humanitarian organizations to mitigate these negative effects. Early identification and treatment of mental health challenges were essential for reducing the adverse impact on job satisfaction and performance. Our study aligned with an Italian study conducted during the COVID-19 pandemic found that healthcare professionals treating COVID-19 patients faced significantly higher risks of stress, burnout and mental health challenges particularly in the most severely affected areas. Notably, twice as many of these professionals considered seeking psychological support compared to those not working directly with COVID-19 patients [18]. A study from China found that anxiety and depression significantly predicted the level of STS in emergency and intensive care nurses, highlighting the importance of addressing these mental health issues to reduce STS among healthcare professionals working in high-stress environments [19].

Quality of life demonstrated a strong protective effect across all job-related outcomes underscoring the value of promoting enhancements in quality of life for aid workers. Improved work-life balance, recreational activities and safer working conditions can contribute to reduced burnout and higher satisfaction levels. This was particularly relevant as a higher quality of life also correlated with lower STS suggesting that structural and policy-based interventions aimed at enhancing quality of life could have a lasting positive impact on aid workers’ mental health and resilience. Overall Quality of Life (QoL) in the context of humanitarian aid workers often reflected the balance between their personal and professional lives [20]. Factors such as social support, family relationships and coping mechanisms can significantly affect their overall quality of life [21]. For instance, strong family support has been associated with lower levels of burnout and greater life satisfaction among aid workers [22]. Additionally, life satisfaction and emotional well-being are crucial components of QoL and can be influenced by various aspects of work-related stress [23].

Stress was strongly associated with burnout and STS, emphasizing the need for tailored stress management interventions for aid workers. Strategies such as resilience training, workload management, and counseling services could help mitigate the risks of burnout and STS in this workforce. Given the inherently high-stress environment of humanitarian work, these interventions are essential for preserving both the mental and physical health of aid workers. Our findings are consistent with those of a Greek study on healthcare professionals, which showed a strong correlation between occupational stress and both burnout and STS indicating that increased stress levels among health professionals are associated with greater ProQoL challenges [24]. Similarly, a study on mental health nurses in Israel found that those who perceived their work as more stressful also experienced higher levels of burnout and lower compassion satisfaction supporting the importance of stress-reduction measures in high-stress professions [25].

Self-esteem not only influenced burnout and compassion satisfaction but also played a significant role in enhancing resilience. Strengthening self-esteem through organizational support, skill-building and positive reinforcement could further improve resilience and overall job satisfaction among aid workers. These strategies can help create a supportive work environment that bolsters workers’ sense of competence and value. Research on healthcare workers had demonstrated that higher self-esteem is associated with lower burnout levels and greater compassion satisfaction [26]. Studies involving mental health professionals had found that low self-esteem correlated with higher burnout and secondary traumatic stress while individuals with higher self-esteem tend to have better coping mechanisms and report greater compassion satisfaction [27].

The study's findings provided strong evidence for targeted interventions to foster a supportive work environment in humanitarian settings. Organizations should consider implementing regular mental health check-ins, stress management workshops and accessible family support resources. Given the strong effect of PTSD on burnout and STS, organizations should also prioritize trauma-specific mental health resources for workers who have been exposed to traumatic events. Finally, it is important to recognize the limitations of the study such as sample size or representativeness which may affect the generalizability of the findings. Future research should focus on evaluating specific interventions designed to reduce burnout and improve psychosocial well-being among humanitarian aid workers. By building on this research, organizations can develop evidence-based strategies that enhance the resilience and overall quality of life of humanitarian staff.

### 4.1 Strengths and limitations

This study has several strengths. It addresses a timely and important topic by exploring professional quality of life and mental health disorders among humanitarian aid workers in Thailand. A large and representative sample was used, enhancing the generalizability of the findings. Validated scales were employed and the questionnaire was pre-tested to ensure measurement validity. The analyses were thorough and appropriate including regression models and correlation analyses to examine associations between key variables. However, the study also has some limitations. The cross-sectional design limits causal inference, as observed associations may be influenced by confounding factors or reverse causality. Data were collected through self-reported measures, which may be affected by social desirability bias and recall bias, potentially impacting the accuracy of the findings. Additionally, the study was conducted in a specific geographic region along the Thai-Myanmar border which may limit the generalizability of the results to other humanitarian contexts.

### 4.2 Conclusion

This study emphasized the significant role of social support, mental health and quality of life in shaping the professional quality of life of humanitarian aid workers. Strong family and social networks were found to enhance compassion satisfaction while reducing burnout and secondary traumatic stress. Conversely, high levels of stress, depression and PTSD were major negative predictors of work satisfaction contributing to higher burnout and distress. This study highlighted the importance of mental health support for humanitarian aid workers who experience moderate levels of compassion satisfaction, burnout and secondary traumatic stress (STS). Strong family and social support were linked to higher job satisfaction and lower burnout emphasizing the need for targeted interventions. Mental health conditions like stress, depression and PTSD significantly contributed to burnout and STS underscoring the necessity of early screening and intervention. Additionally, higher quality of life (QOL) was associated with greater job satisfaction and lower stress levels suggesting that improving work-life balance and overall well-being could enhance resilience. Implementing mental health education, peer support and coping strategies in clinical practice can help sustain the well-being of humanitarian workers ensuring they can continue providing essential services effectively.

## What this study adds

• This study provides insights into the prevalence and impact of mental health disorders among humanitarian aid workers along the Thai-Myanmar border.
• This study assesses the Professional Quality of Life (ProQOL), focusing on compassion satisfaction, burnout, and secondary traumatic stress, offering a comprehensive view of workers' well-being.
• This study identifies key factors such as stress, depression, PTSD, and social support that significantly influence ProQOL outcomes, informing targeted interventions.

## Implications for policy and practice

• The findings highlight the need for policies that address mental health support for humanitarian aid workers, integrating mental health services into organizational frameworks.
• This study suggests the implementation of training programs focusing on stress management and resilience building to enhance workers' coping mechanisms.
• This study recommends allocating resources towards mental health services and support systems to improve the overall well-being and effectiveness of humanitarian aid workers.

## Author statements

Roshan Kumar Mahato, Naw Lar Paw, Kyaw Min Htike and Rajitra Nawawonganun made substantial contributions to the study's conception, design, data acquisition, analysis and interpretation. Roshan Kumar Mahato took the lead in drafting the manuscript while all authors participated in revising it critically for important intellectual content. All authors have read and approved the final version of the manuscript and agreed to be accountable for all aspects of the work ensuring its accuracy and integrity.

## Availability of data and materials

Not applicable.

## Ethics statement

The study received ethical approval from the Centre for Ethics in Human Research, Khon Kaen University, Thailand (Reference No. HE662274).

## Funding

None.

## Declaration of competing interest

The authors declare that they have no known competing financial interests or personal relationships that could have appeared to influence the work reported in this paper.
